# Analysis of energy consumption and change structure in major economic sectors of Pakistan

**DOI:** 10.1371/journal.pone.0305419

**Published:** 2024-07-01

**Authors:** Qianwen Bai, Muhammad Yousaf Raza

**Affiliations:** School of Economics, Shandong Technology and Business University, Yantai, Shandong, China; Hong Kong Shue Yan University, HONG KONG

## Abstract

Studying and analyzing energy consumption and structural changes in Pakistan’s major economic sectors is crucial for developing targeted strategies to improve energy efficiency, support sustainable economic growth, and enhance energy security. The logarithmic mean Divisia index (LMDI) method is applied to find the factors’ effects that change sector-wise energy consumption from 1990 to 2019. The results show that: (1) the change in mixed energy and sectorial income shows a negative influence, while energy intensity (EI) and population have an increasing trend over the study period. (2) The EI effects of the industrial, agriculture and transport sectors are continuously rising, which is lowering the income potential of each sector. (3) The cumulative values for the industrial, agricultural, and transport sectors increased by 57.3, 5.3, and 79.7 during 2019. Finally, predicted outcomes show that until 2035, the industrial, agriculture, and transport incomes would change by -0.97%, 13%, and 65% if the energy situation remained the same. Moreover, this sector effect is the most crucial contributor to increasing or decreasing energy consumption, and the EI effect plays the dominant role in boosting economic output. Renewable energy technologies and indigenous energy sources can be used to conserve energy and sectorial productivity.

## 1. Introduction

Energy has become a fundamental parameter used to regulate a country’s economy [1]. Macroeconomic development relies on the continuous investigation of new energy sources and industrial technology innovation [2]. The industrial, transport and agriculture sectors are considered among the leading sectors that play an imperative role in the growth of the economies and also play a significant role in our daily lives, but unluckily these are also major sources of energy utilization [3]. Because of technical and industrial competition, the energy demand of Pakistan is rising at a higher level than its primary energy supply. These sectors mainly utilize fossil fuel energy that produces a negative impact on the country’s economy and environment. Various sectors (i.e., domestic, commercial, industrial, agriculture, transport, and other government) of Pakistan consumed energy by 59.77 million tons of oil equivalents (Mtoe) [4]; however, industrial, agriculture and transport energy, reached 21.54 Mote, 0.87 Mtoe, and 20.66 Mtoe in 2019, which is about 69% of the overall sectorial energy consumption. These sectors are the significant economic sectors in Pakistan, which have provided a share of gross domestic product (GDP) of 19.74%, 18.74%, and 13.2% [5].

**Fig 1:**
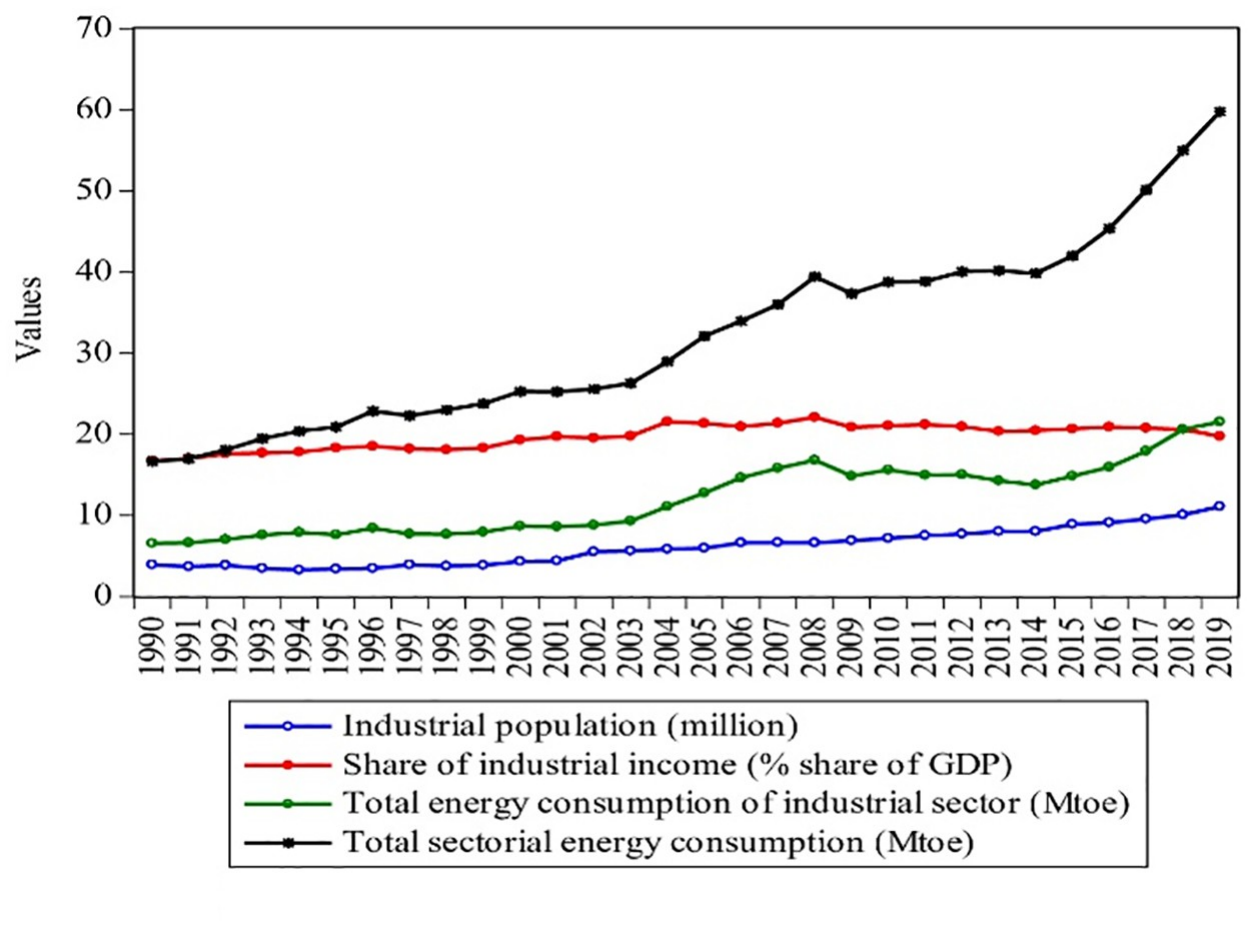
Contribution of industrial sector of Pakistan during 1990–2019. **Source:** [4, 9].

**Fig 2:**
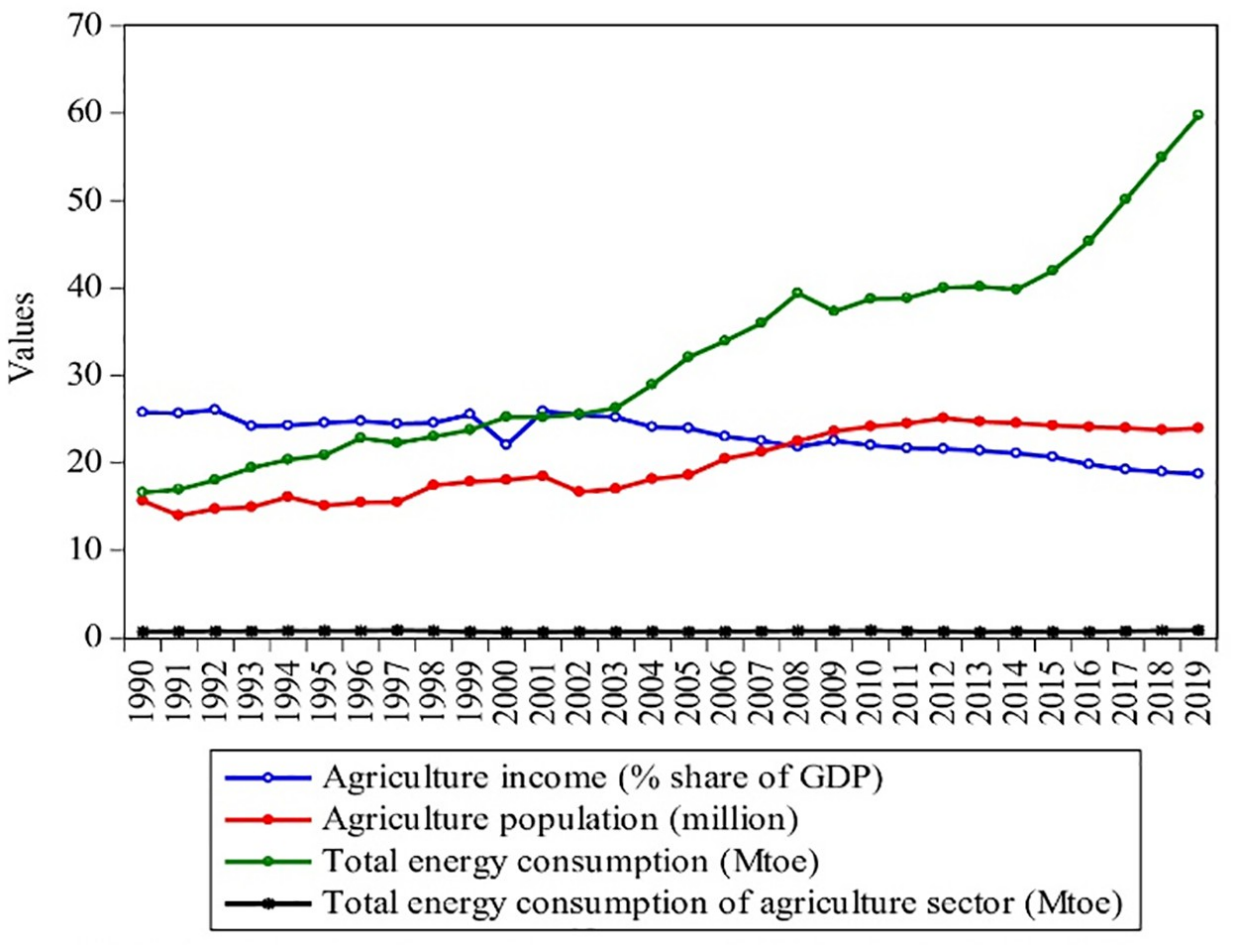
Contribution of agriculture sector of Pakistan during 1990–2019. **Source:** [4, 9].

**Fig 3:**
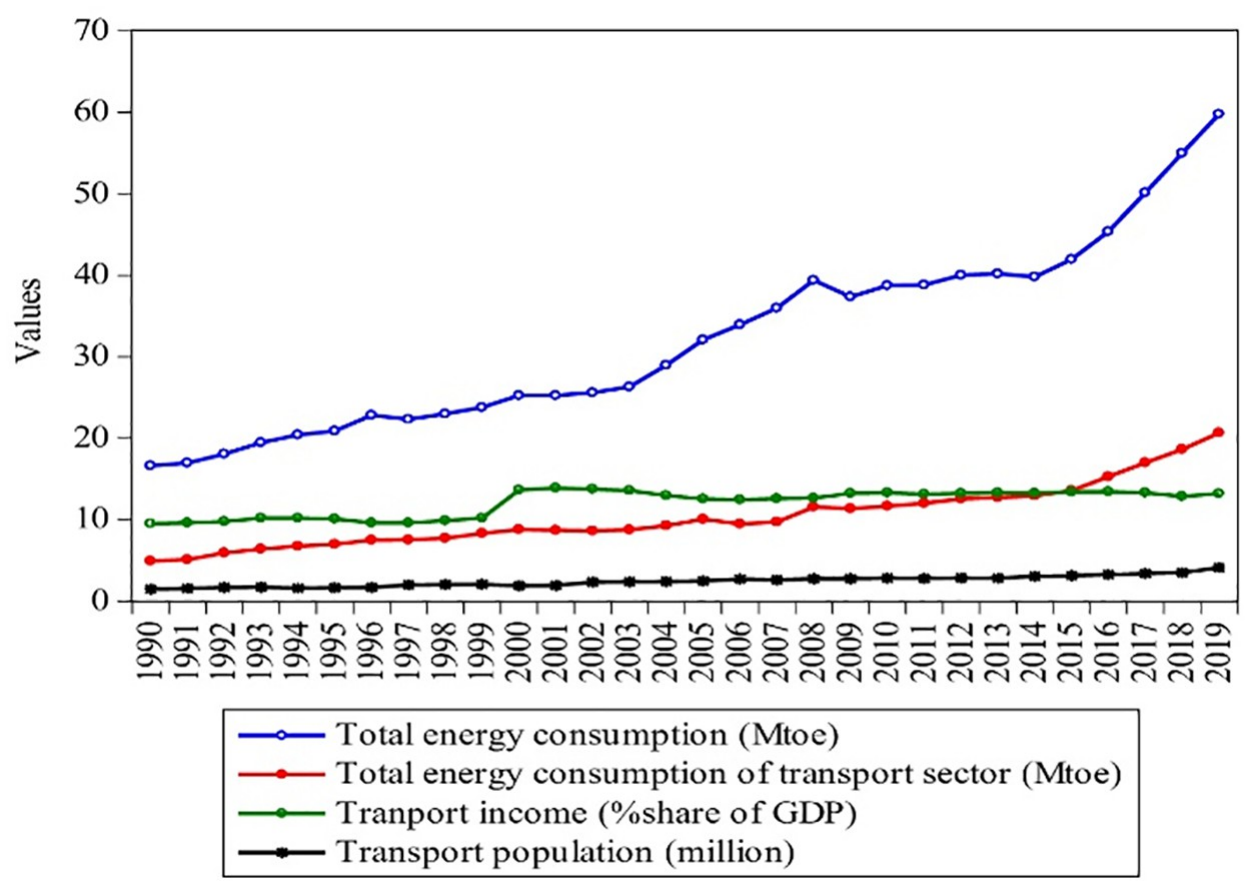
Contribution of transport sector of Pakistan during 1990–2019. **Source:** [4, 9].

Figs 1–3 shows Pakistan’s industrial, agricultural, and transport energy consumption with the rise of their income and population from 1990–2019. With the rapid economic growth, the final energy consumption of all sectors in Pakistan rose from 16.64 Mtoe to 59.77 Mtoe from 1990 to 2019, showing an annual average growth rate of 2.6%. Between 1990 and 2019, industrial energy consumption grew at an annual rate of 2.29%, rising from 6.55 Mtoe to 21.54 Mtoe, as shown in Fig 1. However, with the rise in energy consumption, the share of industrial GDP increased from 16.7% to 20% from 1990–2019, with a population growth of 3.93 million to 11.1 million. The industrial sector is a vast energy consumer and plays a vital role in promoting Pakistan’s coordinated economic development progress. Fig 2 shows that agricultural energy consumption rose at an annual rate of 0.21%, growing from 0.71–0.86 Mtoe from 1990–2019. According to the Pakistan Bureau of Statistics [5], agriculture is an important sector of Pakistan and added 18.74% to the GDP in 2019, as shown in Fig 2. The agriculture sector of Pakistan is the biggest sector, with a maximum population (of 23.98 million), which grew by 0.53% during 1990–2019. It can also be seen that from 1990 to 2019, the share of agriculture income decreased by 0.27%. This is because; the agriculture sector is dependent on electricity and oil and devoid of other energy sources such as coal and gas.

The transport sector plays an essential role in our current society, adding to approximately 20% of the world’s energy use [6]. Road transport, rail, aviation, and shipping are directly or indirectly linked to the various economic sectors and have economic and social effects [7]. This improves the economy and creates a linkage between people, economic paths, regions, and the rest of the world [8]. The transport sector has become a maximum energy-consuming sector, which has added 13.2% to the GDP and employment by 7% in Pakistan [4]. Fig 3 presents that the transport sectors’ energy consumption increased at an annual rate of 3.19%, growing from 4.93 Mtoe to 20.66 Mtoe from 1990–2019. The transport sector of Pakistan is the fastest-growing population sector, which has a maximum population (of 4.1 million), which grew by 1.73% during 1990–2019. It can be confirmed that from 1990–2018, the share of transport income increased by 0.39% due to the addition of petroleum products (i.e., natural gas, CNG, and petroleum products) have been added during the current decade [9]. Overall, it can be estimated that the industry consumes more than 72% of energy, and the transport sector of Pakistan, which are directly associated with the economy of Pakistan.

This determined national goal has activated substantial pressures to lessen carbon dioxide (CO_2_) emissions by using minimum petroleum products in many sectors [8]. The industrial, agriculture and transport sectors are considered the three pillars of energy consumption in Pakistan, accounting for about 43.06 Mtoe out of 59.8 Mtoe in 2019 [9]. Thus, it is essential to ascertain the peaks of enormous energy consumption at the national and sectorial levels. Moreover, it is also necessary to strategically analyze the main driving factors of sectorial energy consumption to attain the objectives on time. Although, in 2019, the world’s energy consumption (i.e., oil, natural gas, coal, nuclear electricity, hydroelectricity, and renewable energy) was counted at 13946.21 Mtoe [10]. However, the world’s top ten energy-consuming economies (i.e., The United States, Canada, France Germany, China, India, Russia, Brazil, Japan, and South Korea), especially in 2019, show a rising share of energy consumption. For this, numerous countries have built policies to lessen fossil fuel consumption and thus added a portion of renewable energy in various sectors [1].

**Fig 4:**
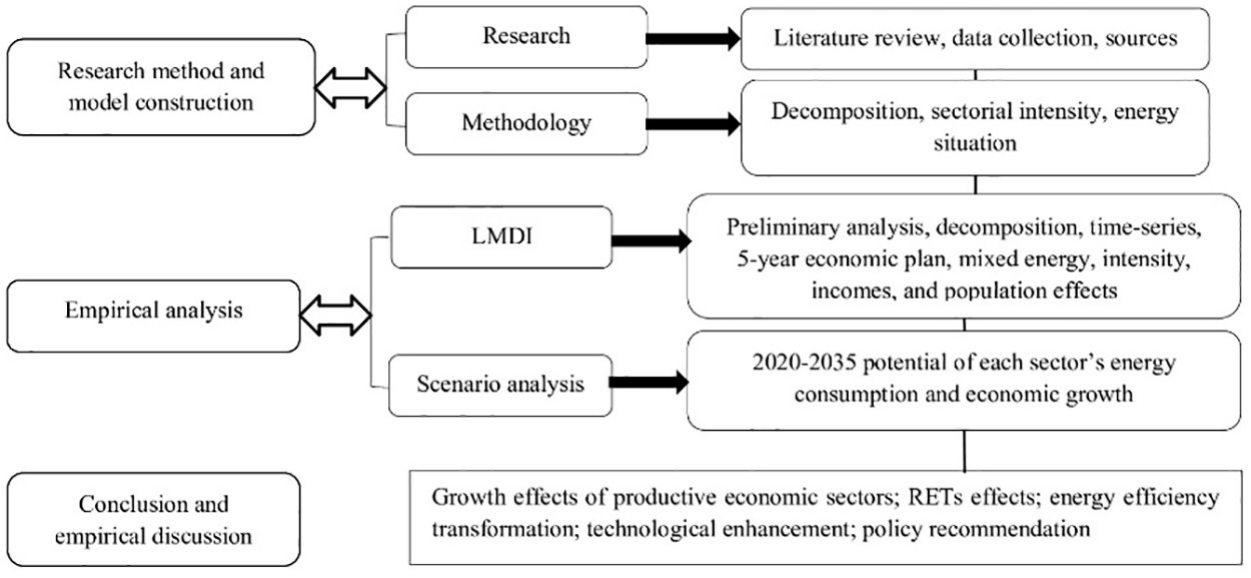
Research model showing energy-related factor’s decomposition of contributing sectors.

This study is concerned with what factors have the most influence on industrial, agricultural, and transport energy consumption in Pakistan. These sectors account for a major share of energy consumption, particularly petroleum products. Pakistan has imported approximately more than 47% of its energy from other countries (Pakistan Economic Survey [4]), directly influencing Pakistan’s economy. Energy utilization is expected to grow due to economic development, population growth, industrialization, urbanization, transportation services, and agriculture growth, which make Pakistan confront two great challenges: (1) oil supply security and environmental challenges; and (2) distribution of energy in various sectors for efficiency. Therefore, Pakistan’s energy security and related policies must analyze the driving forces governing energy use in the existing sectors. Moreover, the determined national goals have triggered substantial pressures on energy security, environmental issues and economic growth in many sectors, in which transport, agriculture and industrial sectors are the main pillars of energy consumption [9]. Hence, it is considered important to identify the energy and economic situation not only at national level factors but also at the sectorial level. Also, it is necessary to investigate the major leading factors in sectorial energy consumption in order to strategically attain the peaking goals on time. Moreover, Fig 4 presents the research model based on sectorial contribution in energy mix, energy intensity, income, population, and future scenario analysis for Pakistan.

The principal contributions of the current study are fivefold. (1) It quantifies the impact of various factors (i.e., sectorial energy mix, energy intensity, income, and population) and delves into the mechanisms through which sectorial accumulation effects a country’s growth. This efficiently fills a gap in current research that has broadly been dependent on econometric models, providing useful direction for Pakistan and other developing economies quick urbanization and industrialization. (2) Different decomposition methods can be applied to accomplish the decomposition analysis between various factors (Ang [11]); however, this study focusses on logarithmic mean Divisia index (LMDI) method. For instance, it is perfect in decomposition and can control multiple factors easily. Two main properties show that (a) there is no unexplainable residual term coming from the decomposition, such as the aggregated energy utilization, which can be attributed to each factor. (b) It allows analysts to add more factors to the analysis. Thus, this property has become more imperative in current times as researchers try to rise the number of factors in a decomposition analysis. The comparisons between LMDI and related decomposition methods can be seen in Ang [11] and Ang and Zhang [12]. Thus, this technique is adopted to investigate the association between the various driving factors [13]. (3) Past studies concentrated on environmental pollution, GDP growth, the effects of CO_2_ emissions, and the growth of a particular sector; for example, Hu et al. [14] analyzed the relationship between energy and economic growth in the agriculture sector of China; Khatun and Ahamad [15] investigated the foreign direct investment and energy relationship for Bangladesh’s power sector; Raza et al. [3] analyzed the relationship between energy, economy and pollution in the transportation sector of United States. These studies have focused in terms of emerging or developed economies with the relationship between energy, economy and environmental issues at sectorial levels employing the different methods (i.e., decomposition and regression analysis). However, these studies are very limited in nature and concentrated on individual sectors or limited sectors. Thus, this study is conducted to the best of our knowledge that found the substitution effects of various factors in the major economic-contributing sectors in Pakistan. (3) Under the limited period, the estimation is divided into six sub-periods, i.e., 1990–1994, 1995–1999, 2000–2004, 2005–2009, 2010–2014, and 2015–2019 to give policies at five-year economic planning of Pakistan (5-year plan information can be obtained from here. https://sites.google.com/site/maeconomicsku/home/economic-planning-in-pakistan.), which will help government policy makers to investigate exactly targeted estimations. The reason for selecting Pakistan for this analysis is; Pakistan consumes about 69% of its energy consumption for transport, industrial and agriculture sectors [9]. Although very little analysis has been seen in the industrial, agriculture, and transport sectors individually based on the LMDI method. This analysis can give practical and wide-ranging recommendations for the government for expressing energy security, energy substitution and climate change. Fourth, the study can be extended to other countries and answer the following questions: (i) What are the key contributing factors within the framework of the industrial, agriculture and transport sectors? What are the effects of sectorial energy intensity, income, and population during the planning period? (iii) What is the prediction of factors until 2035 under Pakistan’s visions 2025–2035 scenarios? Finally, based on the LMDI analysis and fitting line of forecasting from 2020–2035, the research advises energy policies for the selected sectors.

The remaining part of the study is as follows: section 2 provides the literature review. Section 3 discusses method and section 4 provides data information. Section 5 provides the empirical results and discussion are provided in section 6. Conclusion and policy recommendations are provided in section 7.

## 2. Literature review

Past studies in the field of energy use connection have mostly concentrated on the association between energy and related indicators, for instance, economic development, CO_2_ emissions and country-levels [16–19]. The standard technique to estimate the energy utilization between various sectors in an economic system is the input-output method. This method is based on ‘3’ parts, including backward, forward and overall linkages [20–22]. They estimated the linkage impact between certain sectors to compare the overall variations of the economic system.

In addition, different studies have seen different variables that impact energy use in various countries and multiple industries, sectors and timeframes. For example, Kulu et al. [23] for Sub-Saharan African countries using the General Methods of Moments; Xu et al. [24] for China’s energy and environmental perspectives in various sectors; Raza and Lin [25] for Pakistan’s factor productivity under energy and economic growth; Raza and Tang [26] for Pakistan’s nuclear energy, economy and CO_2_ emissions; Yu [27] for desirable capital flows in the emerging economies. Nevertheless, many researchers paid attention to the issue of CO_2_ emissions from various sectors, including industry, agriculture, and transport. For example, Chen et al. [28] analyzed the carbon-intensive divisions (i.e., industrial, building, transport, and agriculture) of China; Hao et al. [29] examined the CO_2_ emissions based on Chinese provinces; Ouyang and Lin [30] analyzed CO_2_ from China’s industrial sector; Alshehry and Belloumi [31] analyzed the CO_2_ emissions from the transport sector of Saudi Arabia; Chen et al. [32] investigated the CO_2_ emissions in the construction industry of China, Li et al. [33] estimated the energy-related CO_2_ emissions in the agriculture sector of the European Union, and Raza and Hasan [34] concentrated on Bangladesh’s manufacturing and industrial sector’s carbon emissions. In addition, Shao et al. [35] analyzed the green technology innovation with CO_2_ emissions using the cross-sectional augmented autoregressive distributed lags method for the N-11 countries. They found that green technology and renewable energy have a negative long-run relationship with CO_2_ emissions. Liu et al. [36] analyzed the relationship between energy, CO_2_ emissions and carbon-intensity in China using dynamics simulation. They estimated that in various economic growth rates, energy and CO_2_ emissions in 10% GDP growth. Thus, sustainable energy substitution and technological progress are significant in reducing pollution [37]. According to Wang et al. [38], turbulence can affect the generation of energy by influencing the efficiency and stability of various energy production processes, such as wind and hydroelectric power, where fluctuating fluid dynamics can enhance or disrupt the capture and conversion of kinetic energy into electricity. These researchers used different methods, such as the autoregressive distributive lag model, decomposition analysis, input-output method, turbulence modeling, etc. to investigate the various factors. They concluded that economic growth is the most significant factor in influencing whether energy is utilized maximally or minimally.

Currently, different decomposition approaches have been applied to estimate the impact of various factors on the variation of energy use and various sectors, such as regression analysis, structural decomposition analysis (SDA), index decomposition analysis (IDA), and so on [39–41]. Two well-known decomposition methods, namely SDA and IDA, have been broadly applied to examine the driving forces in the literature. Rose and Casler [42] analyzed that SDA is based on the input-output (I-O) model in quantitative economics based on its theoretical review and major features. Furthermore, many researchers used this method in quantitative research; for example, Wood [43] used SDA for greenhouse gas emissions in Australia; Kagawa and Inamura [44] for Chinese and Japanese energy demand; Chang et al. [45] for Taiwan’s industrial CO_2_ emissions; Liu et al. [46] for China’s energy embodied in international trade, and Zeng et al. [47] for China’s energy intensity. Ang and Zhang [12] and Sun [48] described the two types of IDA: the Laspeyres decomposition analysis and the Divisia index decomposition analysis. Each IDA can be used in a period or in a time-series way. A period-wise analysis compares indices between the first and last year of a given country, industry, region, etc. Though, the outcomes of a period-wise decomposition are susceptible to the choice of a base year and final year, it does not express how the impacts of the decomposition factors have changed over the studied time. The time-series analysis included yearly decomposition using time-series data, and its outcomes illustrate how the effects of pre-defined independent factors have changed over time. Anyway, period-wise results can be found from a time-series analysis, but not vice versa. Though, there is no consensus among them on whether this is the best decomposition approach [46]. Ang [39] compared many IDA techniques and concluded that the LMDI approach was the favored method because of its theoretical foundation, adaption, convenience of application, outcome interpretation, and desirable properties decomposition analysis. Thus, we applied the LMDI method to examine Pakistan’s industrial, agriculture, and transport sectors from 1990–2019, identifying the driving factors and analyzing the corresponding contributions to sectorial-related energy consumption.

Thus, this method is applied to investigate the relationship between sectorial energy consumption, energy intensity, income and population effects from Pakistani perspectives. At the local-level, we found that many of the previous studies have employed different methods with different factors, thus, they did not give particular proof of sectorial energy and their socio-economic factors from Pakistani perspectives, which is different from previous studies. Also, the literature exposed that the future trend in energy substitution is unclear, which gives insight into the internal mechanisms of Pakistan’s energy consumption, which has become more imperative in the current decades. This presents the theoretical and practical implications of sectorial energy research.

Thus, from the Pakistani perspective, only few studies have estimated the association between energy ingestion, but most of them found the relationship between CO_2_ emissions and energy consumption in different sectors, for example, Lin and Raza [8] examined the energy-related CO_2_ emissions from the power sector; Lin and Ahmad [7] analyzed energy substitution effect of transport sector of Pakistan by using trans-log production function; Raza and Lin [49] examined the CO_2_ emissions from the transport sector by using logarithmic mean Divisia index (LMDI) and decoupling approach; Zaman et al. [50] examined the relationship between agriculture technology and energy demand; Mushtaq et al. [51] measured the cointegration and causality analysis for Pakistan’s agriculture sector; Mirza et al. [52] analyzed the China-Pakistan economic corridor relationship with energy use and energy saving by applying cointegration analysis, and Biresselioglu et al. [53] examined the household energy use by national survey. Though, within the context of Pakistan, most of the relevant studies paid attention to the individual sectors or at a national level rather than the sectorial level. Thus, many studies related to significant energy-consumption and economic growth sectors are absent.

Based on the above analysis, we estimate that energy utilization in different sectors is incomplete due to method application, time-frame, and factor selection. The decomposition method was employed in this study, which added to finding the energy utilization roles in all sectors of Pakistan. Considering major productive sectors in Pakistan as an example, this study estimated and investigated the energy consumption movements in sectors over 1990–2019 on the availability of maximum information. The exact pathways regarding energy flows in the major sectors were investigated to suggest more targeted policies for energy conservation and value-added. Moreover, new policies, social changes, and renewable energy substitution will lead to bottlenecks and competition within the network [54]. As per the energy and socio-economic factors, it is necessary to investigate the sectorial efficiency. The employed factors and their substitution are imperative for sectorial planning, energy use, energy technologies, and social development in Pakistan.

## 3. Methodology

### 3.1. Energy-related decomposition model

The logarithmic mean Divisia Index (LMDI) can be demonstrated as an extended Kaya identity by Kaya [55], while Ang [39] found that the LMDI technique is based on the Kaya identity and is the optimal one due to its unmatched advantages in the theoretical foundation, ease of use, and result in interpretation. Following the present research, among IDA (index decomposition index), the LMDI is preferable for its path independence, accumulative consistency, and ability to handle ‘0’ values [56–58]. The LMDI can be applied to calculate CO_2_ emissions, energy efficiency, energy consumption structure, and energy intensity, for instance, Lin and Raza [8], Raza and Lin [49], Wang et al. [59], and Raza [60] used the decomposition method to check the energy, socio-economic and CO_2_ emission factors for China and Pakistan and suggested that more differentiated policies are needed to be implemented in various economic sectors to mitigate the carbon emissions. Technical measurement, alternatively, is another aspect that is growing the productivity of energy to substitute fossil fuels and carbon mitigation potential. For this, the LMDI method was generally employed to investigate the factors that impact energy use and carbon emission reduction [17]. As an important energy source, there is a degree of substitution between energy, the economy, and related factors such as mixed energy, energy intensity, and socio-economic elements. However, the energy substitution effect and internal structure should not be overlooked when analyzing sector-specific energy factors. Given its applicability, ease of use, interpretability, and solid theoretical foundation, this method is utilized to construct the decomposition analysis in the current study. Consequently, LMDI is generally used in making policy insights into, for example, the drivers of energy utilization (i.e., Lin and Raza [21], Ma and Stern [61] and Zhang and Guo [62] and changes in carbon emissions [28, 63–65]. The LMDI estimation can be utilized to compare a series of indices and discuss their effects on the trends during the period from the base year (zero) to the current year (1) [8, 61]. Moreover, a study by Wang [66] found that this model can be used to predict environmental issues. For this purpose, the study applies the LMDI that permits the perfect disaggregation of variations into driving forces, whose contributions can be added to the overall utilization variation without residual terms. It is a broad employed method used in different scientific research organizations and agencies (Ó Broin et al. [67]), and its advantages have been intensely studied in the literature [58]. In the existing research, Pakistan’s sectorial energy-related consumption (i.e., industrial, agriculture and transport) is estimated from 1990 to 2019. Moreover, using the LMDI method, we obtain the increase in energy consumption of entire industries caused by an individual impact factor from 1990–1994, 1995–1999, 2000–2004, 2005–2009, 2010–2014, and 2015–2019. The effects of each sector on its total energy consumption cannot be overlooked because the nominated sectors are Pakistan’s primary economic sectors. Therefore, the existing research decomposes changes in energy consumption based on four decomposition factors (i.e., energy mixed, energy intensity, income, and sectorial population effects). The objective is to estimate the effects of each factor based on each sector in Pakistan. In this study, Pakistan’s sectorial energy consumption (*SEC*^*t*^) is decomposed as follows:

$$SEC^{t}=\sum_{j}\;\sum_{k}\;\sum_{l}\;\frac{TEC_{jkl}^{t}}{TEC^{t}}\times \frac{TEC^{t}}{I_{jkl}^{t}}\times \frac{I_{jkl}^{t}}{P^{t}}\times P_{jkl}^{t}$$ (1)

$$SEC^{t}=\sum_{j}\;\sum_{k}\;\sum_{l}\;ES_{jkl}^{t}\times EI_{jkl}^{t}\times I_{jkl}^{t}\times P_{jkl}^{t}$$ (2)

where *SEC*^*t*^ is the energy consumption of the industrial, agriculture and transport sector; j, k and l show the total energy consumption of industrial, agriculture, and transport sectors. $ES_{jkl}^{t}$, $EI_{jkl}^{t}$, $I_{jkl}^{t}$, and $P_{jkl}^{t}$ are the mixed energy, energy intensity, income, and population effects of sectors *j*, *k* and *l*. Also, factors in Eqs (1) and (2) are further described in Table 1. Thus, based on Eq (1), $ES_{jkl}^{t}$ is characterized by four factors, as follows in Eq (2):

1. $ES_{jkl}^{t}=\frac{TEC_{jkl}^{t}}{TEC^{t}}$ indicates the mixed energy (*j*, *k* and l) effects, which show the changes in the relative shares of energy forms in total energy consumption of sectors.
2. $EI_{jkl}^{t}=\frac{TEC^{t}}{I_{jkl}^{t}}$ indicates the energy intensity of fuel type *j*, *k* and l. This estimates the changes in the energy consumption ratio to the net income of given sectors.
3. $I_{jkl}^{t}=\frac{I_{jkl}^{t}}{P^{t}}$ shows the income effect changes in each sectors’ per capita net income.
4. $P_{jkl}^{t}$ indicates the population effects in the given sectors, which shows the changes in the total population of each sector.

**Table 1:** LMDI formulae for sectorial energy consumption [39, 58].

IDA Identity	$SEC^{t}=\sum_{j}\;\sum_{k}\;\sum_{l}\;\frac{TEC_{jkl}^{t}}{TEC^{t}}\times \frac{TEC^{t}}{I_{jkl}^{t}}\times \frac{I_{jkl}^{t}}{P^{t}}\times P_{jkl}^{t}$	
Change structure	For sectorial factor’s effects based on [0,t] Ang and Liu [57] can be estimated as. $\Delta SEC=SEC(1)-SEC(0)=\Delta SEC_{ES_{jkl}}+\Delta SEC_{EI_{jkl}}+\Delta SEC_{I_{jkl}}+\Delta SEC_{P_{jkl}},$ $\begin{array}{ll} SEC(1) & =SEC(0)+{\left(\Delta SEC_{ES_{jkl}}\right)}_{effect}+{\left(\Delta SEC_{EI_{jkl}}\right)}_{effect}+{\left(\Delta SEC_{I_{jkl}}\right)}_{effect}\\ & +{\left(\Delta SEC_{P_{jkl}}\right)}_{effect}, \end{array}$ While, SEC (0) can be written as. $\begin{array}{ll} SEC(0) & =SEC(1)-{\left(\Delta SEC_{ES_{jkl}}\right)}_{effect}-{\left(\Delta SEC_{EI_{jkl}}\right)}_{effect}-{\left(\Delta SEC_{I_{jkl}}\right)}_{effect}\\ & -{\left(\Delta SEC_{P_{jkl}}\right)}_{effect}, \end{array}$	
	Driving factors	Symbols
	industrial, agriculture and transport sectors	*jk*l
	Sectorial energy consumption	*SEC* ^*t*^
	Mixed energy	$ES_{jkl}^{t}$
	Energy intensity	$EI_{jkl}^{t}$
	Income	$I_{jkl}^{t}$
	Population	$P_{jkl}^{t}$
Sectorial effects	Effects by using LMDI formulae	
	Effects of using LMDI formulae	
Mixed energy effects	$\Delta SEC_{ES_{jkl}\;\text{effect}}=\sum_{j}\;\sum_{k}\;\sum_{l}\;W.\;\text{ln}\left(\frac{ES(1)}{ES(0)}\right)$	
Energy intensity effects	$\Delta SEC_{EI_{jkl}\;\text{effect}}=\sum_{j}\;\sum_{k}\;\sum_{l}\;W.\;\text{ln}\left(\frac{EI(1)}{EI(0)}\right)$	
Income effects	$\Delta SEC_{I_{jkl}\;\text{effect}}=\sum_{j}\;\sum_{k}\;\sum_{l}\;W.\;\text{ln}\left(\frac{I(1)}{I(0)}\right)$	
Population effects	$\Delta SEC_{P_{jkl}\;\text{effect}}=\sum_{j}\;\sum_{k}\;\sum_{l}\;W.\;\text{ln}\left(\frac{P(1)}{P(0)}\right)$	
	Where, $W=\left(\frac{SEC(1)-SEC(0)}{\text{ln}(SEC(1)-SEC(0))}\right)$ in which SEC (1) − SEC (0) measures the energy consumption at the current and base year.	

**Note:** Δ presents the change.

Thus, the individual sector’s decomposition can be estimated in Table 1.

## 4. Data sources

The present research tests the LMDI model in Pakistan at the sectorial level based on the availability of time-series data from 1990 to 2019. In this study, we used country-level data analysis. Due to the absence of provincial and individual industrial levels, they have not been used; thus, the investigation is based on all the major energy-economic contributing sectors. The data for the present research came from three various sources. The first is the Pakistan Energy Yearbook [9], which contains energy-related data from selected sectors. The study is modeled based on four factors. Each sector’s energy is different and aggregated based on consuming energy resources. For example, in Pakistan, the industrial sector consumes oil, gas, electricity, and coal; the agriculture sector consumes oil and electricity; and the transport sector consumes ten kinds of fuels, including aviation fuel, motor spirit, HOBC, E-10, kerosene, HSD, LDO, furnace oil, electricity, and natural gas. All the fuel data has been collected from the Pakistan Energy Yearbook [9] and the Pakistan Economic Survey [4]. All the energy-related data, for instance, in transport, industrial, and agriculture, has been collected in million tons of oil equivalents (Mtoe). Using these data with Eq (1), each sector’s energy situation can be estimated. The second is the Pakistan Economic Survey [4], which gives each sector’s GDP and population share (million) to test the income and population effects. The third is the Pakistan Bureau of Statistics [5], which provides the sectorial share of GDP at constant basic prices (% share of GDP). The objective is to see the individual contribution of each sector. A detailed description of each variable with their average and standard deviation is presented in Table 2. From Table 1, each factor’s effect is measured based on the 5-year interval. The related sector’s data has been collected and arranged based on Five-Year Plans, i.e., 1990–1994, 1995–1999, 2000–2004, 2005–2009, 2010–2014, and 2015–2019. The Five-Year Plan of Pakistan is arranged based on the Planning Ministry, which is convenient for exploring the results. https://sites.google.com/site/maeconomicsku/home/economic-planning-in-Pakistan. Lin and Raza [8, 21] and Raza and Lin [49] have applied a similar interval plan based on the Pakistan Bureau and the National economic plans of Pakistan. The employed variables, such as mixed energy, energy intensity, income, and population effects of sectors are important and state the strength and effects. On the basis, we employed the LMDI method to calculate the changes in different sectors and energy use in Pakistan which can be used to check the economic condition. Thus, the current variables are important to investigate their association because Pakistan is an emerging economy and most of its economy depends on fuel [37].

**Table 2:** Description of variables.

Variables	Unit	Sources	Mean	Std. Dev.	Definition
Industrial energy consumption	Mtoe	[4, 5]	12.0277	4.4041	It is the overall energy consumption (i.e., oil, coal, gas, and electricity) in the industrial sector, including manufacturing, mining and quarrying, large- and small-scale industries.
Agriculture energy consumption	Mtoe	[4, 5]	0.7538	0.0576	Total energy consumption, including oil and electricity in the agriculture sector. All the crops, livestock, fishing, tractors, and forestry are included.
Transport energy consumption	Mtoe	[4, 5]	10.3532	3.8720	Total use of energy, including aviation fuel, motor spirit, HOBC, kerosene, HSD, LDO, furnace oil, electricity, and natural gas. Transport related to trade, storage and communication.
Industrial income	% share of GDP	[5]	19.7171	1.5219	Industrial income includes the mining and quarrying, large scale, small scale, slaughtering, electricity generation, gas distribution, and construction industries are included.
Agriculture income	% share of GDP	[5]	23.0817	2.2267	Agriculture incomes come from the forestry, fishing, important crops, other crops, cotton ginning, and livestock.
Transport income	% share of GDP	[5]	12.0840	1.6327	Transport income come from the overall communication, logistics, storage, and trade.
Industrial population	Million	[4, 5]	6.0687	2.2816	It is the total people linked with the industrial sector.
Agriculture population	Million	[4, 5]	19.8400	3.8860	It is the total people linked with the agriculture sector.
Transport population	Million	[4, 5]	2.4493	0.6680	It is the total people linked with the transport sector.

## 5. Empirical findings

### 5.1. The results of the LMDI model and relative indicators

Using the equations in Table 1, the effects and contribution of individual factors on the sectorial energy consumption of j, k, and l are described in Table 3. As shown in Table 3, the population factor generally plays a major driving effect for the rise in energy consumption; the energy mixed effect has an increasing trend in the industrial and transport sectors, while the agriculture sector shows a decreasing trend in the current phase. Sectorial income has a negative impact due to the mixed energy effect. Furthermore, yearly sectorial energy consumption results with their coefficient of variation growth from 1990–2019 and forecasted increase/decrease of each factor are deliberated in detail in the next section.

**Table 3:** Energy-related factor’s decomposition of contributing sectors.

Sector	Year	** $\Delta SEC_{j_{effect}}$ **	** $\Delta SEC_{EI_{j\;effect}}$ **	** $\Delta SEC_{II_{j\;effect}}$ **	** $\Delta SEC_{IP_{j\;effect}}$ **	Total effects
Industrial	1990–1994	-1.5749	15.4288	27.6049	-20.5849	20.8739
	1995–1999	-10.1309	15.4272	-14.4860	14.4826	5.2928
	2000–2004	16.7758	4.0882	-28.9591	45.3522	37.2571
	2005–2009	0.0210	36.5098	-35.2268	30.5142	31.8182
	2010–2014	-33.9870	12.3517	-30.9063	24.5198	-28.0219
	2015–2019	5.2791	109.6557	-73.6715	60.9787	102.2419
	1990–2019	-16.8939	213.7623	-167.5155	199.6728	229.0257
		$\Delta SEC_{IP_{\text{k}\;effect}}$	$\Delta SEC_{EI_{\text{k}\;effect}}$	$\Delta SEC_{AI_{\text{k}\;effect}}$	$\Delta SEC_{AP_{\text{k}\;effect}}$	Total effects
Agriculture	1990–1994	-9.9349	27.8894	-9.2635	2.9193	11.6103
	1995–1999	-23.8731	9.5271	-13.2566	17.4707	-10.1319
	2000–2004	-5.2289	4.8731	8.0980	0.5952	8.3374
	2005–2009	-5.9256	22.6170	-32.0047	25.4502	10.1369
	2010–2014	-20.7395	7.7347	-6.5617	1.7697	-17.7967
	2015–2019	-15.1165	49.4627	-9.6030	-1.3181	23.4252
	1990–2019	-6.8303	10.0520	-4.6826	2.6713	1.2104
		$\Delta SEC_{ES_{\text{l}\;effect}}$	$\Delta SEC_{EI_{\text{l}\;effect}}$	$\Delta SEC_{TI_{\text{l}\;effect}}$	$\Delta SEC_{TP_{\text{l}\;effect}}$	Total effects
Transport	1990–1994	12.8161	15.5946	0.7609	7.5724	36.7440
	1995–1999	6.6885	18.5165	-34.4883	35.9974	26.7140
	2000–2004	-14.9776	34.5137	-55.7597	46.2541	10.0305
	2005–2009	-6.4812	21.3905	-10.2279	21.6463	26.3277
	2010–2014	19.2735	7.2218	-21.7585	21.1579	25.8946
	2015–2019	22.6126	125.9889	-99.7421	94.5810	143.4405
	1990–2019	34.2314	211.4839	-150.6586	223.8693	318.9260

***Note*:** j,k and l represent the industrial agriculture and transport sectors.

***Source***: Author’s estimation based on model formula.

## 6. Discussion

After getting the outcomes from the LMDI method, the discussion is carried-out sector by sector.

### 6.1. Analysis of the proportioned impact of the industrial sector’s traditional energy resources and efficiency

In the last decade, Pakistan has broadly relied on imported energy (i.e., coal, oil, gas, and electricity) rather than preferred indigenous energy resources [1]. The industrial sector is tremendously intensive and accountable for 39.5% of total energy consumption in Pakistan [4]. The industrial sector and Pakistan consume oil, gas, coal, and electricity, which have increased by approximately 15% in 2019. Energy consumption of Pakistan’s industrial sector effects from 1990–2019 are presented in Table 3, which is divided into six phases. The components of the industrial decomposition analysis, i.e., $\Delta SEC_{ES_{j}\;\text{effect}}$(energy mix), $\Delta SEC_{EI_{j}\;\text{effect}}$(energy intensity), $\Delta SEC_{I_{j}\;\text{effect}}$(industrial income), and $\Delta SEC_{PI_{j}\;\text{effect}}$(industrial population) effects are based on Table 1, respectively. From 1990–1994, 1995–1999, and 2010–2014 energy mix affected change with a decrease of 1.575, 10.131, and 33.987 Mtoe. During 2000–2004, 2005–2009, and 2015–2019, the mixed energy effect changed to a maximum level of 16.756 Mtoe, 0.021 Mtoe, and 5.279 Mtoe. During the last phase, 2015–2019, the energy mix increased by 15.53% compared to the previous phase. The overall energy mixed effect changes during 1990–2019 decreased by 16.894 Mtoe, which shows that the influence of mixed energy in the industrial sector of Pakistan was not significant. Also, Lin and Raza [21] estimated the industrial structure and industrial energy by using the LMDI approach in Pakistan and found that industrial energy consumption is increasing and mixed energy and energy intensity are growing in the current phase, 2014–2018. The only factor that leads to energy consumption is energy intensity. This is because Pakistan relies on all kinds of energy resources [1, 9].

From 1990 to 2014, the change in industrial income was 27.605 Mtoe/%share. In all other phases, including 1995–1999, 2000–2004, 2005–2009, 2010–2014, and 2015–2019, the industrial income factor decreased to an increasing level of 14.496, 28.959, 35.226, 30.906, and 73.671 Mtoe/%share. This is because of the energy supply and energy import because the industrial sector consumes the maximum energy that comes from imported oil, gas, and electricity. Moreover, unskilled labor has decreased the efficiency of industrial productivity; it has raised energy consumption and polluted the environment, which is consistent with the study of Xiuhui and Raza [68]. Also, there are numerous inconsistencies, for instance, huge energy demand, uncontrolled population, insufficient energy production, circular debt, technical losses, high prices, and cyclical cuts in hydropower are the main impacting factors on the efficiency of industrial value-added [4]. Thus, the growing energy prices for non-renewable energy reduce carbon emissions because of the substitution effect; however, the gross domestic product rises the pollution, which can be strengthened by lowering the non-renewable energy prices [69]. According to the Pakistan Energy Yearbook [9], the overall imported petroleum products decreased by 11.9% with an increase in oil imports (a bill of US$ 11.9 billion), as compared to the previous year. Thus, due to the massive dependency on imported fuel consumption and bills, the industry is unable to manage its potential. Overall, during 1990–2019, the industrial income decreased by 167.515 Mtoe/% shares, which is evidenced by the last four phases. From the perspective of the industrial population during 1990–1994, industrial population effects changed by -20.585 million. During 1990–2019, the population was the essential driving factor for increasing energy and the economy. At the same time, this factor has a mixed and positive trend in all phases, and the overall population has increased by 199.673 million during 1990–2019. The instability is due to the structure, which has not changed yet. To maintain the rapid growth of the industrial economy and the GDP of Pakistan, the country should focus on the huge reserves of coal, oil, hydel power, and renewable energy technologies (RETs). Finally, based on all factors, the industrial effects changes are found to be positive except for phase-4, 2010–2014. The GOP should enhance the industrial structure, which has not converted the whole sector from fossil fuels to renewables and advanced technologies. To make a significant improvement in efficiency and economy, the industrial structure should be based on all kinds of fuels, particularly the domestic fuel reserves, which are in line with Lin and Raza [1, 21] and Chen et al. [28].

### 6.2. Analysis of the proportioned impact of the agriculture sector’s traditional energy resources and efficiency

Being an agricultural country, this sector’s energy mix needs more energy for the production and economic growth of Pakistan. Currently, the agriculture sector of Pakistan is only dependent on oil and electricity, which consumed 2.12% of the total energy consumption of industrial, agriculture and transport sectors Pakistan Energy Yearbook [9]. Energy consumption from the various factors of Pakistan’s agriculture sector (i.e., $\Delta SEC_{ES_{k}\;\text{effect}}$, $\Delta SEC_{EI_{k}\;\text{effect}}$, $\Delta SEC_{I_{k}\;\text{effect}}$, and $\Delta SEC_{P_{k}\;\text{effect}}$) from 1990–2019 are provided in Table 3. From 1990–1994, 1995–1999, 2000–2004, 2005–2009, 2010–2014, and 2015–2019, the oil and electricity effects decreased by 9.935, 23.873, 5.229, 5.926, 20.739, and 15.116 Mtoe. Overall, in 30 years (from 1990–2019), the oil and electricity consumption in agriculture decreased by 16.894 Mtoe, which estimates that the influence of mixed energy in the agriculture sector was not significant. Due to the decreasing trend of mixed energy in the agriculture sector, the agriculture income was also showing a decreasing trend in most phases, such as 1990–1994, 1995–1999, 2005–2009, 2010–2014, and 2015–2019 by 9.263, 13.256, 32.005, 6.562, and 9.603%share/million. During 2000–2004, the agriculture income effect was estimated to increase by 8.098%share/million.

The energy intensity ($\Delta SEC_{EI_{k}\;\text{effect}}$) is the only factor showing a rising trend. This expresses the total energy consumption per unit change in income of the agriculture sector. As per Lin and Raza [8], if the energy intensity is small, the energy consumption will be more efficient. All the phases given in the $\Delta SEC_{EI_{k}\;\text{effect}}$ provide an increasing trend, which confirms that the energy mix in the agriculture sector is not efficient. Overall, it can be seen that the energy intensity changed by 10.052 Mtoe/%share from 1990–2019. Furthermore, the agriculture sector of Pakistan is the biggest population concerned sector, and half of the employed labor force of Pakistan is directly/indirectly associated with this sector Pakistan Bureau of Statistics [5]. Based on the analysis, most of the phases, including 1990–1994, 1995–1999, 2000–2004, 2005–2009, and 2010–2014, show a trend of increasing effects of 2.919, 17.471, 0.595, 25.450, and 1.769 million. While the current phase, 2015–2019, shows a decreasing effect of 1.318 million, which is due to the dependence on only two energy sources, i.e., oil and electricity. To improve agriculture efficiency, increase employment, and improve the economy of the country, the GOP should encourage the farmers to provide the RETs and should add other fuels such as gas and biogas to the agriculture sector. In addition, the agriculture sector is the lifeblood of Pakistan because half of the population is employed by Raza et al. [70], and this sector is the largest source of foreign exchange earnings. A lot of deficiencies have been seen in industrialization, which needs a huge number of substitutive resources in the future [71]. For instance, recently, the government is considering estimations to give motivations of 19.3 billion to farmers for growing agriculture-related products.

### 6.3. Analysis of the proportioned impact of the transport sector

The transport sector of Pakistan is one of the important sectors, contributing 13.2% to the GDP and employing 3% of the country in 2019 [4]. This sector is based on many related activities (i.e., trade, services, natural resources, community, and society). Thus, this sector has become the second huge energy-consuming sector in Pakistan, having consumed more than 34% of total energy in 2019 [4, 9]. It is noted that recently transport sector relies now only on aviation fuel, motor spirit, HOBC, E-10, kerosene, HSD, LDO, furnace oil, electricity, and natural gas. Besides, due to the huge energy crisis in the last decade, Pakistan could not maintain its energy supply in all sectors. Thus, the transport sector could not continue its electricity consumption from 2012 to-date [4, 9]. Table 3 gives the energy consumption from the various factors of Pakistan’s transport sector from 1990–2019, which are $\Delta SEC_{ES_{l}\;\text{effect}}$, $\Delta SEC_{El_{l}\;\text{effect}}$, $\Delta SEC_{I_{l}\;\text{effect}}$, and $\Delta SEC_{P_{l}\;\text{effect}}$. During 1990–1994, 1995–1999, 2010–2014, and 2015–2019, the energy mixed effect increased by 12.816, 6.688, 19.273, and 22.613 Mtoe, while $\Delta SEC_{ES_{l}\;\text{effect}}$ was decreased only in two phases (2000–2004 and 2005–2009) with a huge amount of 14.977 and 6.481 Mtoe. Overall, during 1990–2019, the transport mixed energy effect changes were analyzed by 34.231 Mtoe, which showed significant results. The $\Delta SEC_{EI_{l}\;\text{effect}}$ and transport population effects are the only factors that have a positive trend in all phases, which are increasing by 211.483 Mtoe/%share and 223.869 million during 1990–2019. As shown in Table 3, the energy intensity effects of the transport sector are high, showing that energy consumption is not efficient. The outcomes are in line with the industrial and agriculture sectors and also in line with the assumptions [8, 21]. Because of the low energy supply, high energy intensity, and huge population, the transport income showed a decreasing trend in all phases, excluding 1990–1994. During 1990–1994, the maximum transport income was 0.761%share/million, while the maximum decrease in income during 2015–2019 was estimated as 99.742%share/million. Overall, the income effect of the transport sector from 1990 to 2019 was estimated at -150.658%share/million, which is not supportive of the economic growth of Pakistan. Finally, the GOP should add its share of electricity and renewable energy in the transport sector of Pakistan, which has been discussed by Raza and Lin [49]. Furthermore, the Pakistan Energy Yearbook [9] reported that Pakistan’s imported electricity and renewable energy were added by 1.1% and 0.1% to the primary energy supply, respectively. Therefore, this energy share should be added to the transport sector to enhance income and employment. Also, transport is the backbone that has added 6% and 10% to the employment and economic growth of Pakistan [71]. The changes, current productivity and future potential present significant productivity, energy security and climate change due to indigenous and worldwide cooperation, Pakistan and China are trying to spread their trade, defense, economic situation, and energy security under the China-Pakistan Economic Corridor, One-Belt-One-Road. Furthermore, new vehicles have become a trend, which can give reference potential pattern for other countries like Pakistan, whose market for new energy vehicles is in the early stages [72]. This is imperative because the World Bank highlighted Pakistan’s efforts to enhance energy, economy and environment [73]. Thus, Pakistan should reduce oil imports, substitute domestic energy and enhance technological development for future targets, which will not only boost output but also enhance economic and environmental sustainability.

### 6.4. Effects of estimated factors based on energy consumption and cumulative growth

**Fig 5:**
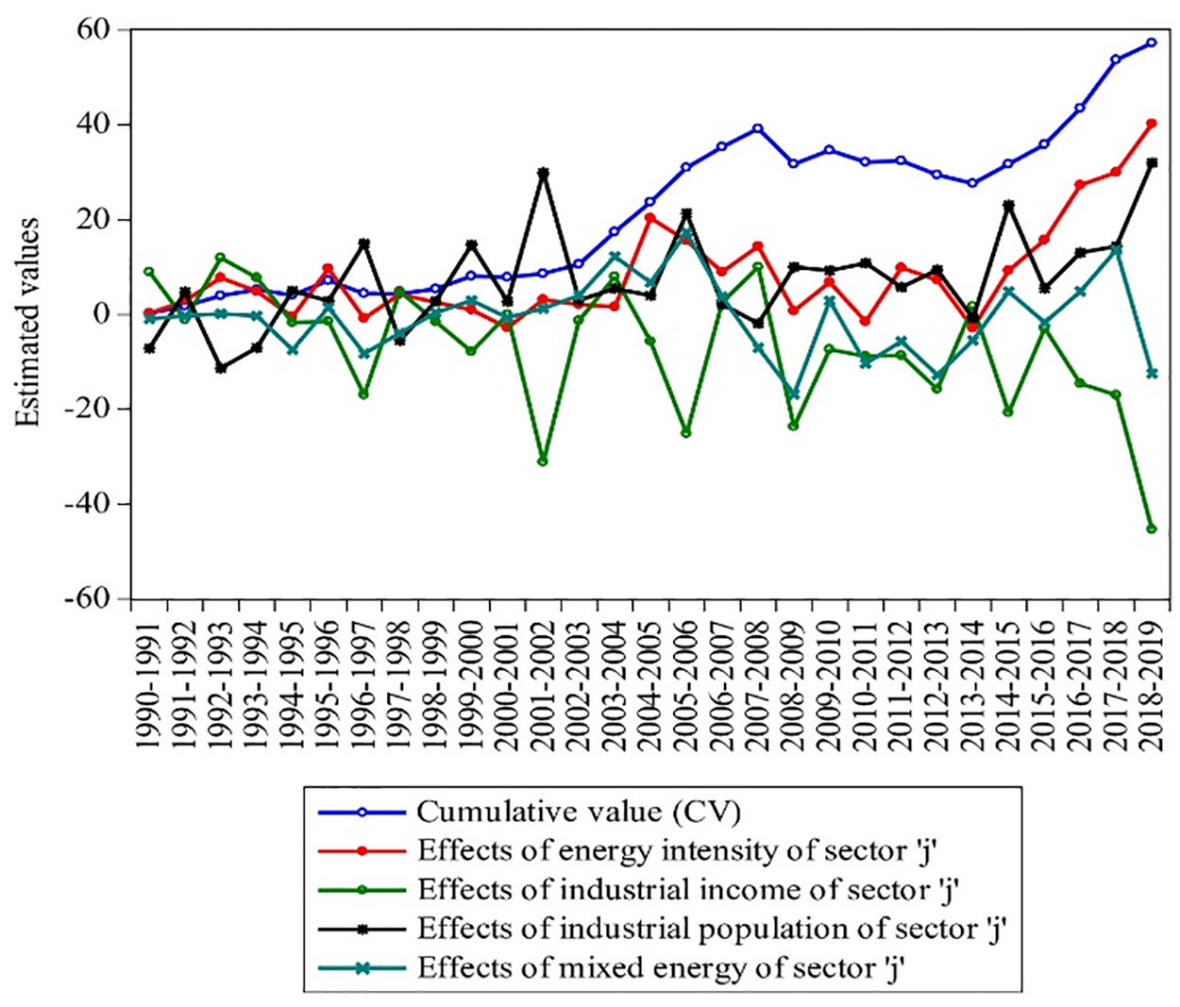
Industrial energy consumption effects with CV growth from 1990–2019. **Source:** [4].

**Fig 6:**
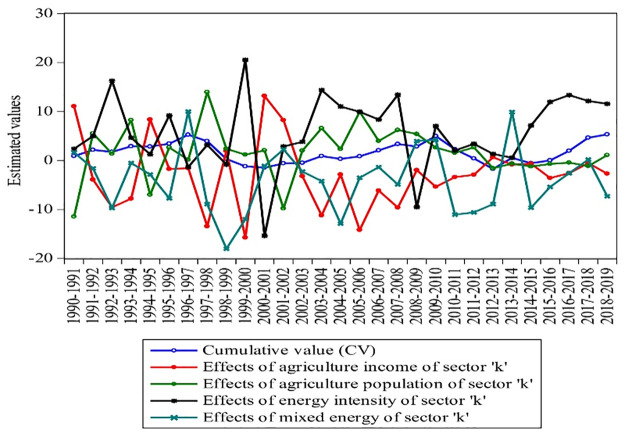
Agriculture energy consumption effects with CV growth from 1990–2019. **Source:** [4].

**Fig 7:**
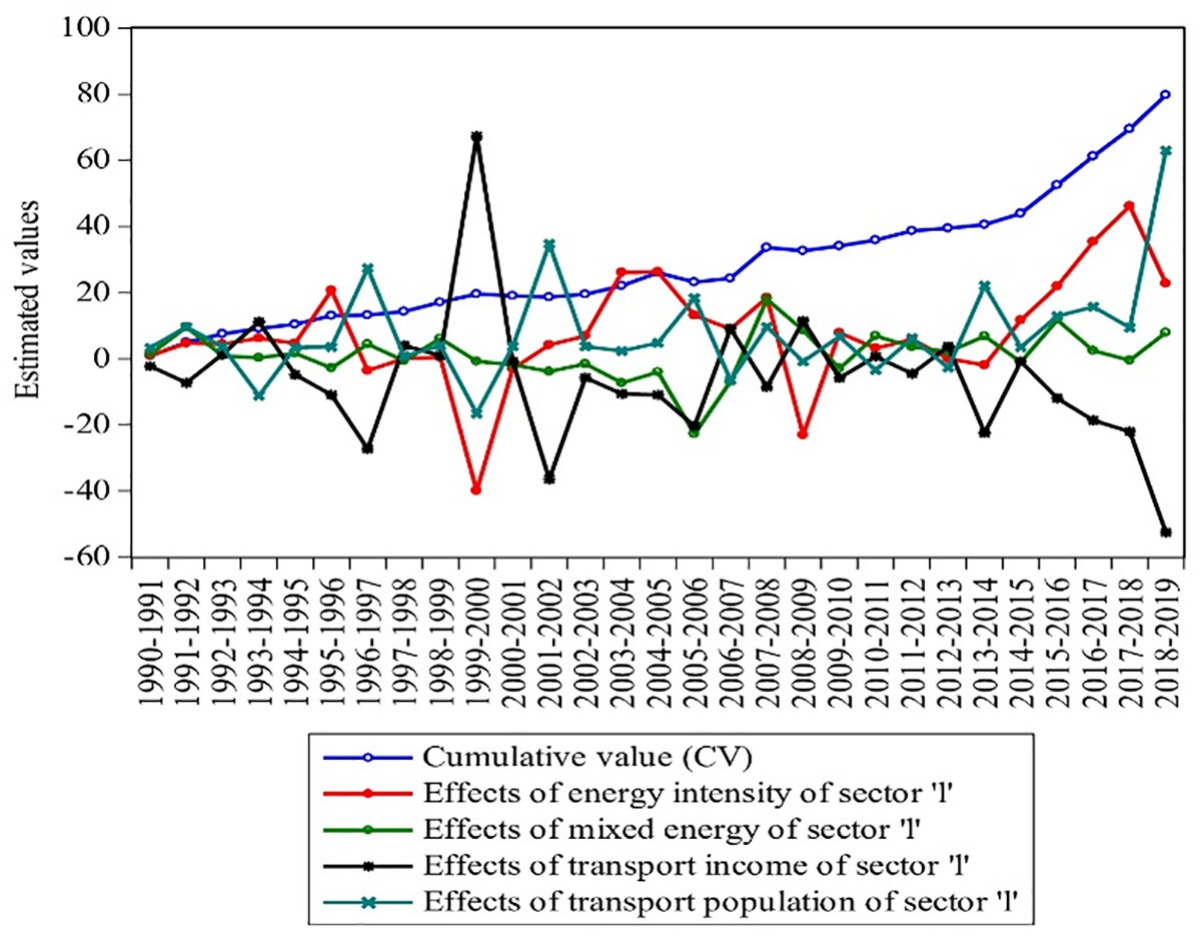
Transport energy consumption effects with CV growth from 1990–2019. **Source:** [4].

This subsection will deliberate the different driving factors with their annual effects. $ES_{jkl}^{t}$, $EI_{jkl}^{t}$, $I_{jkl}^{t}$, and $P_{jkl}^{t}$ estimate the mixed energy, energy intensity, income, and population of Pakistan’s industrial, agricultural, and transport sectors. Each factor’s cumulative value (CV) is also figured with individual variations in each year, as shown in Figs 5–7.

From Fig 5, it can be seen that the overall mixed energy growth of the industrial sector (j) remained decreasing from 1990 to 2019 periods by 16.892 Mtoe, but the mixed energy was growing from 1993, 1996, 1999–2000, 2002–2007, 2010, 2015, and 2017–2018. It is also clear that due to the lack of mixed energy and high energy intensity, the industrial income remained negative by -167.509%share/million during 1990–2019. The industrial income remained positive only in 1991, 1993–1994, 1998, 2004, 2007–2008, and 2014 because of the small energy intensity, which is in line with the study of Lin and Raza [21]. Finally, the industrial population remained positive and was counted at 199.670 million during 1990–2019. It has also been estimated that population effects remained to decrease by a small ratio in most energy crisis periods, such as 1991, 1993–1994, 1998, 2008, and 2014. Overall, in the last decade, $ES_{j}^{t}$, $EI_{j}^{t}$, $I_{j}^{t}$, $P_{j}^{t}$, and CV fluctuated by -26%, 56.38%, 91%, 2.19%, and 81%, which shows the expansion of the driving factors (i.e., economic scale, the industrial revolution, mixed energy, and energy intensity) have a strong effect on industrial energy. This was linked to the rise in economic growth in Pakistan [1]. Also, the reduction in the economy is due to a lack of technology, and industry dropped due to alternative energy resources and the energy crisis [74]. Finally, Pakistan’s energy consumption during 2019 shows that the CV rose by 57.26 times.

Fig 6 estimates the $ES_{k}^{t}$, $EI_{k}^{t}$, $I_{k}^{t}$, $P_{k}^{t}$, and CV of the agriculture sector from 1990–2019. Mixed energy effects were found to be negative from 1990–2019 by -119.774 Mtoe. The increasing trend has been estimated in 1993, 1997, 2002, 2009–2010, 2014, and 2018 by 1.63, 10, 2.25, 3.96, 4.22, 9.88, and 0.21 Mtoe. The overall, energy intensity remained increasing due to the energy crisis and also had a negative impact on agricultural income. It can be seen in Fig 6 that the $EI_{k}^{t}$ and $I_{k}^{t}$ changed by 176.31 Mtoe/%share and 228.183%share/million from 1990 to 2019, which shows a decreasing impact on the agriculture economy. The agricultural population increased by 46.85 million during 1990–2019. However, the population increased by 0.97 million in 2019, which confirms that agricultural labor is increasing and can play an imperative role in the growing economy. From the perspective of CV, Pakistan’s energy consumption during 2019 shows that CV increased by 5.32 times and 88.21 times during 1990–2019. In the long-run, investment and exports have been the two main drivers of economic growth in Pakistan’s agriculture sector. However, many studies have shown the energy demand and technological effects of the agriculture sector; for example, Aqeel and Butt [75] estimated the relationship between agriculture and the economic growth of Pakistan. He also analyzed that energy consumption leads to agricultural technology. Similarly, Von Braun [76] evidenced the effects of technological change in agriculture production in Western Africa; Bonny [77] estimated the French energy intensity in the agriculture sector and found that agriculture seems to have become more efficient in its use of energy; Schroll [78] for the Danish agriculture sector, who analyzed that energy output increase agriculture production, and Ozkan et al. [79] for Turkish agriculture and examined how the total input of energy increased output in agriculture production. Finally, it can be seen that in the last decade, $ES_{k}^{t}$, $EI_{k}^{t}$, $I_{k}^{t}$, $P_{k}^{t}$, and CV changed by -283.95%, -221.73%, 36.99%, -79.56%, and 86.72%, which shows that the agriculture energy use affects agriculture income.

Fig 7 presents the transport sectors’ $ES_{k}^{t}$, $EI_{l}^{t}$, $I_{l}^{t}$, $P_{l}^{t}$, and CV from 1990–2019. The energy mixed structure of the transport sector had a major influence on the whole study period. The mixed energy ($ES_{l}^{t}$) reduces 2.79, 0.67, 0.82, 1.81, 3.89, 1.59, 7.35, 4.05, -22.77, 6.86, 2.99, 1.03, and 0.45 Mtoe in 1996, 1998, 2000–2007, 2010, 2015, and 2018, respectively. The minor impact might be attributed to fuel supply mostly occurring between aviation oil, E-10, kerosene, LDO, furnace oil, and electricity, which are declining over time [9]. Besides, the $EI_{l}^{t}$ effects were high with 211.48 Mtoe/%share during 1990–2018 and presented a negative impact on the transport income, which is counted at 150.63%share/million. The transport-related population increased by 223.86 million during the whole study. The employed population declined by 11.19, 16.67, 6.55, 0.84, 3.42, and 2.71 million in 1994, 2000, 2007, 2009, 2011, and 2013. It can be evidenced from the studies of Imran [80], Raza and Lin [49], and Lin and Raza [74], that most transportation consumes petroleum and natural gas products, while oil products are the largest consuming products in this sector. Furthermore, it can also be noted that population and economic growth decreased by 75.33% and 5.59% in the last decade, which can be improved by increasing the energy mix. The cumulative sum of the transport sector shows that the CV rose by 79.73 times during 2019 and 159.47 times during 1990–2019. Overall, it can be estimated that $ES_{l}^{t}$, $EI_{l}^{t}$, $I_{l}^{t}$, $P_{l}^{t}$, and CV fluctuated by -0.08%, -1.98%, -5.59%, -75.33%, and 1.44% in the last decade of the study.

### 6.5. Sectorial energy and economic growth scenarios

**Fig 8:**
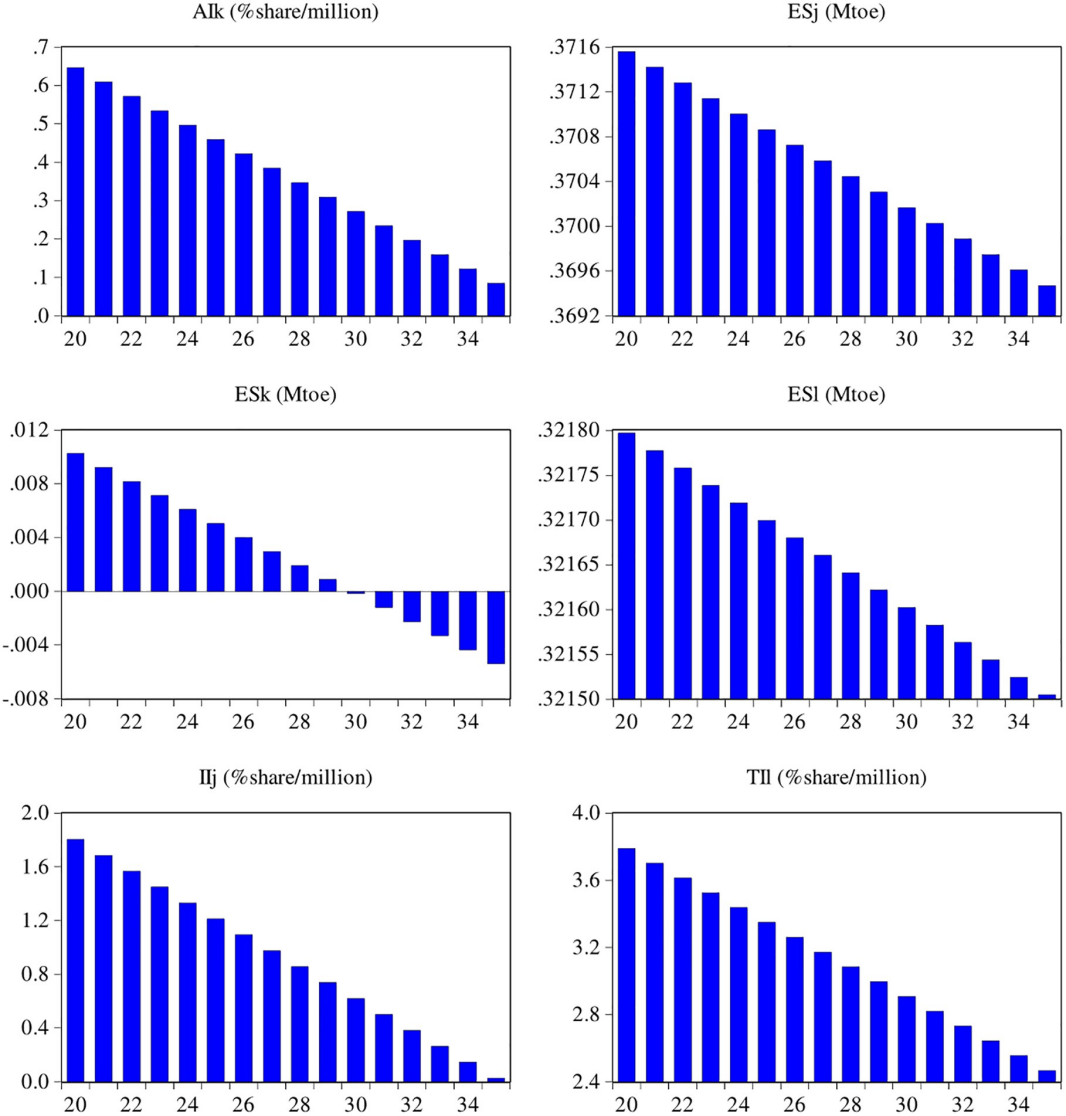
The predicted potential of each sector’s energy consumption and economic growth from 2020–2035. **Source:** [4].

Based on the previous fitting and outcomes of the effects of the equations in Table 1, the predicted fluctuations between energy consumption and economic growth are presented in Fig 8. To analyze the forecasted energy consumption and income of Pakistan’s industrial, agriculture, and transport sectors, we have predicted values for the period of 2020–2035 based on previous studies by Lin and Raza [8] and Raza and Lin [49] for Pakistan.

Using the output in Fig 8, the outcomes of the energy consumption of $ES_{j}^{t}$ have a declining trend from 2020 to 2035. The mixed energy consumption of the industrial sector of Pakistan would decrease by 0.630% during the period. The income level of industrial ($I_{j}^{t}$) would also decrease by 0.973% during the study period, which shows that by declining the energy supply in the industrial sector, the income level would also decline because Pakistan’s economy is mostly dependent on the maximum imported energy. According to the Pakistan Energy Yearbook [9], the annual final energy consumption rate increased by 9.7% in 2018 due to the major increase in industry, agriculture, and transport sectors. This would improve the economic position of the country in the future. It can also be seen from Fig 8 that agricultural economic output is playing a pivotal role in promoting the economy, while mixed energy is declining at the worst level from 2020 to 2035. Overall, it can be concluded that energy consumption in the agriculture sector would decrease by 53%, while the income would remain positive by 13%. This is because half of Pakistan’s employment is associated with agriculture, and solar energy could also be helpful for farmers in the future. From the transport point of view, the mixed ($ES_{l}^{t}$) would decline by 67% during 2020–2035, while the economy would remain positive by 65%. This is because the transport sector is an emerging and huge energy-consuming sector in Pakistan [49, 74].

## 7. Conclusion and policy recommendations

### 7.1. Conclusion

To analyze the energy consumption of the industrial, agriculture, and transportation sectors, we first estimate the sector-wise energy consumption from 1990 to 2019, and then investigate the present status of the sector-wise energy consumption. The logarithmic mean Divisia index (LMDI) method is applied to find out the nature of factors that influence the changes in the selected sectors at the interval of 5-years and each year during the study period. At last, based on the LMDI method, we also predicted comparing sectorial energy consumption with the income from 2020 to 2035. The main conclusions drawn from the existing study can be summarized as follows:

First, under the 5-year national economic plan, the decomposition analysis showed that the energy intensity, value-added and population are the only factors that lead to energy consumption. These results are supported by the literature and mainly added by the contribution of the mixed energy effect. The industrial mixed energy consumption decomposition found a significant output, especially in 2000–2004, 2005–2009, and 2015–2019 planning periods. The overall industrial mixed energy was decreased during the studied period due to income, industrial skills and renewable energy technologies (RETs). However, population and energy intensity show the significant effects in the current planning period, which presents that income potential of the country can be further enhanced by the energy and labor.

Second, the agriculture mixed energy shows a reduction in overall consumption, which has impacted income; however, energy intensity and population effects presented a rising trend during the studied period. This suggests that energy intensity and mixed energy growth directly lead to the economic growth of the agriculture sector in Pakistan. Moreover, the empirical applications to yearly energy inputs over the period. As given in our application, the current model presents that factor substitution is positive, excluding income. This conclusion provides a vision that energy helps as a material for Pakistan’s economy and thus contributes to the value-added nexus.

Third, the transport sector is undergoing fast growth with high energy consumption, and hence increased the mixed energy consumption. The mixed energy was found to increase consumption in the current phase and as a whole with which all the remaining factors have significantly contributed during the studied period. Moreover, the annual analysis describes that the mixed energy, energy intensity and population effects were the major driving factors influencing changes in overall energy consumption. The variation in energy intensity and population factors are the major cause of growing productivity and have future implications, which shows that the intensity effect in Pakistan’s transport sector increased and affected the economic status of the sector.

Finally, based on annual estimation, the cumulative growth of the industry, agriculture, and transport sectors significantly increased during 2019. Based on 2020–2035, the industrial income would decline by 0.97%, and agriculture, and transport sectors would rise by 13%, and 65%, respectively. This is because most of the population of Pakistan is primarily linked to these sectors. Thus, based on the above outcomes, the strategic measures of sustainable industrial, agriculture, and transport policies should aim to improve energy efficiency and strengthen each sector’s income level. These policies are as follows:

### 7.2. Policy suggestions

1. To enhance the effect of EI on mixed energy consumption, the GOP should issue total energy consumption control standards, such as industrial, agriculture, and transport sector efficiency, standards, and vehicle technology, which are the powerful motivation to limit the EI, make energy-saving measures more specific in these sectors, and ultimately enhance energy efficiency. In addition, to improve the industrial economy, efficiency, technological progress, and technical labor, the government must restructure the industrial sector in both small and medium-level industries. For example, a study by Liu et al. [36] for China’s renewable energy and mitigation potential target suggested that promoting the renewable growth is effective in mitigating CO_2_ emissions and alternatively, will sustain the socio-economic development target. In addition, financial subsidies, tax deduction and exemption policies, special feed-in-tariff policies, and technological support are useful for quick implications. Thus, due to the huge demand for fossil fuels, CO_2_ emissions would change the global temperature and definitely impact the economic and living standards of people, which will need energy substitution possibilities in the future. Regarding the transportation sector, which has added 6% to employment and 10% to the economy of Pakistan, fossil fuel energy consumption can be controlled by increasing energy prices, taxes and subsidies at the micro level. Due to the huge fossil fuel supply in this sector, less energy-consuming engines, and renewable energy resources should be applied. However, the agriculture sector can be fully converted to solar, wind and hydroelectricity. This will enhance the environment, capital, labor, and reduce the energy crisis. In order to approach the standard, the political achievement of the local government should be set as a criterion. In addition, the government should increase the research and development investments in all sectors to promote the application of advanced and energy-efficient RETs, such as hybrid engines, for energy substitution in the machinery of these sectors.
2. The sectorial structure could be set and adjusted to enhance the transformation of energy utilization, for instance, industrial, transport and agriculture sectors show high internal energy use impact, and their reliance must fix its sectorial framework, hasten the transformation of energy use, and grow the consumption of cleaner energy resources (based on financial budget). It is essential to optimize the energy structure to depend on huge energy sources (indigenous resources, i.e., coal, oil, gas, and renewables) instead of huge imports. Increasing the cost of fuels could trigger the externalities of energy, such as the cost of storage, society, and the country’s economy. Pakistan pays a high cost to import energy products; for example, in 2018, Pakistan paid a bill of US$ 11.9 for imported petroleum products [9]. Thus, it is suggested that the economy could be stabilized only if the indigenous resources were used. For this, the GOP should strictly follow the latest energy plans, i.e., the China-Pakistan Economic Corridor (CPEC), Vision 2025 [81], and Vision 2035 [82], for the further improvement of energy efficiency. Regarding solar energy, Pakistan has 6–7 sunshine hours/day (Raza et al. [83]), which can be utilized for energy security because it is a renewable energy source and can be utilized anywhere in the world [84]. In addition, due to the significant population growth, the energy-related frameworks in various major contributing sectors must be redesigned to create consciousness among consumers and industrialists.

Furthermore, this research has only two limitations that require future endeavors. This study focused on the huge energy-consuming sectors based on their income and labor, while this study has not estimated the carbon emission peaks. The other limitation is that the cost of mixed energy and importing countries and individual payments to each country has been left for further research. In addition, future research will add more factors to the qualitative analysis of energy and carbon-related driving factors.

## Supporting information

S1 Appendix (XLSX)

S1 File (DOCX)

## Abbreviations

$EI_{jkl}^{t}$: energy intensity effects of sectors i, j and k
$I_{jkl}^{t}$: income effects of sectors i, j and k
$ES_{jkl}^{t}$: mixed energy effects of sectors i, j and k
$P_{jkl}^{t}$: population effects of sectors i, j and k
CO_2_: carbon dioxide
CPEC: China-Pakistan Economic Corridor
CV: cumulative value
EI: energy intensity
GDP: gross domestic product
GOP: Government of Pakistan
IDA: index decomposition analysis
LMDI: logarithmic mean Divisia index
Mtoe: million tons of oil equivalent
RETs: renewable energy technologies
SDA: structural decomposition analysis
SEC^t^: sectorial energy consumption
Δ: difference
