# Supplementary material for: Analysis of energy consumption and change structure in major economic sectors of Pakistan
Source: PLoS One. 2024 Jul 1;19(7):e0305419. doi: 10.1371/journal.pone.0305419 (PMC11216625; doi:10.1371/journal.pone.0305419)
Supplement: S1 File — (DOCX) [file pone.0305419.s002.docx]

**Appendix**

**Appendix A**

Industrial decomposition and factor variations.

| Time | $\Delta SEC_{j_{effect}}$ | $\Delta SEC_{EI_{j\text{ }effect}}$ | $\Delta SEC_{II_{j\text{ }effect}}$ | $\Delta SEC_{IP_{j\text{ }effect}}$ | CV |
| --- | --- | --- | --- | --- | --- |
| 1990-1991 | -0.98743 | 0.18109 | 8.94175 | -7.15255 | 0.24571 |
| 1991-1992 | -0.33028 | 2.88637 | -1.11485 | 4.72335 | 1.78686 |
| 1992-1993 | 0.17160 | 7.74973 | 11.94564 | -11.31466 | 3.92494 |
| 1993-1994 | -0.33496 | 4.85065 | 7.70839 | -7.04207 | 5.22044 |
| 1994-1995 | -7.45554 | -0.49019 | -1.70047 | 4.98421 | 4.05495 |
| 1995-1996 | 1.48568 | 9.65222 | -1.51555 | 2.84543 | 7.17189 |
| 1996-1997 | -8.22730 | -0.87126 | -16.97536 | 14.96362 | 4.39432 |
| 1997-1998 | -3.94476 | 4.29123 | 4.85841 | -5.50540 | 4.31919 |
| 1998-1999 | 0.39300 | 2.54511 | -1.51951 | 2.83150 | 5.38171 |
| 1999-2000 | 2.95425 | 0.94949 | -7.82966 | 14.64213 | 8.06076 |
| 2000-2001 | -0.68546 | -2.85388 | -0.02157 | 2.72437 | 7.85163 |
| 2001-2002 | 1.24439 | 3.10956 | -31.19246 | 29.90479 | 8.61820 |
| 2002-2003 | 4.00145 | 2.08769 | -1.28985 | 2.98021 | 10.56307 |
| 2003-2004 | 12.23380 | 1.62488 | 7.98349 | 5.42774 | 17.38055 |
| 2004-2005 | 6.74544 | 20.32670 | -5.70884 | 4.01186 | 23.72434 |
| 2005-2006 | 17.27493 | 15.51594 | -25.17653 | 21.32103 | 30.95818 |
| 2006-2007 | 3.68738 | 8.96784 | 2.61870 | 2.10330 | 35.30249 |
| 2007-2008 | -7.04379 | 14.37504 | 10.00542 | -1.87537 | 39.16781 |
| 2008-2009 | -16.90216 | 0.70045 | -23.72996 | 10.01524 | 31.68871 |
| 2009-2010 | 2.89850 | 6.81141 | -7.37549 | 9.26172 | 34.58774 |
| 2010-2011 | -10.34267 | -1.54428 | -8.82384 | 10.81183 | 32.11300 |
| 2011-2012 | -5.69845 | 9.81261 | -8.65558 | 5.72259 | 32.40829 |
| 2012-2013 | -12.76844 | 7.27347 | -15.77339 | 9.38482 | 29.43741 |
| 2013-2014 | -5.43832 | -2.89871 | 1.74515 | -0.80112 | 27.58916 |
| 2014-2015 | 4.81411 | 9.22574 | -20.70512 | 23.04257 | 31.68348 |
| 2015-2016 | -1.73088 | 15.69846 | -2.88519 | 5.48477 | 35.82527 |
| 2016-2017 | 4.89153 | 27.25321 | -14.61748 | 13.00601 | 43.45859 |
| 2017-2018 | 13.59733 | 29.94456 | -17.07738 | 14.37754 | 53.66911 |
| 2018-2019 | -12.42890 | 40.19188 | -45.38826 | 31.97801 | 57.25729 |
| 1990-2019 | -16.89213 | 213.76071 | -167.50948 | 199.67003 | 114.51457 |

**Appendix B**

Agriculture decomposition and factor variations.

| Time | $\Delta SEC_{IP_{\text{k }effect}}$ | $\Delta SEC_{EI_{\text{k }effect}}$ | $\Delta SEC_{AI_{\text{k }effect}}$ | $\Delta SEC_{AP_{\text{k }effect}}$ | CV |
| --- | --- | --- | --- | --- | --- |
| 1990-1991 | 1.63076 | 2.39136 | 11.06912 | -11.46447 | 0.90669 |
| 1991-1992 | -1.61484 | 4.98573 | -3.89485 | 5.53368 | 2.15912 |
| 1992-1993 | -9.71943 | 16.26480 | -9.52946 | 1.37922 | 1.75790 |
| 1993-1994 | -0.50040 | 4.64838 | -7.78603 | 8.23681 | 2.90760 |
| 1994-1995 | -2.87012 | 1.25974 | 8.36214 | -6.99392 | 2.84706 |
| 1995-1996 | -7.72909 | 9.19308 | -1.73389 | 2.64541 | 3.44093 |
| 1996-1997 | 10.00462 | -1.32131 | -1.57959 | 0.15133 | 5.25470 |
| 1997-1998 | -8.90572 | 3.19172 | -13.46137 | 13.94358 | 3.94675 |
| 1998-1999 | -18.01607 | -0.81699 | 1.92406 | 2.39394 | 0.31798 |
| 1999-2000 | -11.98572 | 20.54823 | -15.73763 | 1.20360 | -1.17489 |
| 2000-2001 | -1.09875 | -15.36530 | 13.18344 | 2.07333 | -1.47671 |
| 2001-2002 | 2.25558 | 2.84123 | 8.24507 | -9.77241 | -0.58435 |
| 2002-2003 | -2.24618 | 3.83019 | -3.18944 | 2.03250 | -0.47758 |
| 2003-2004 | -4.18687 | 14.37308 | -11.21088 | 6.58989 | 0.91373 |
| 2004-2005 | -12.89877 | 11.00282 | -2.85121 | 2.33983 | 0.31189 |
| 2005-2006 | -3.51375 | 9.93472 | -14.17984 | 9.95405 | 0.86069 |
| 2006-2007 | -1.35552 | 8.37352 | -6.14080 | 4.00300 | 2.08074 |
| 2007-2008 | -4.86450 | 13.42276 | -9.62317 | 6.22797 | 3.37150 |
| 2008-2009 | 3.96786 | -9.50421 | -1.96696 | 5.40966 | 2.84809 |
| 2009-2010 | 4.22872 | 7.02236 | -5.35751 | 2.66019 | 4.98653 |
| 2010-2011 | -11.04120 | 2.05051 | -3.38430 | 1.55140 | 2.28063 |
| 2011-2012 | -10.58215 | 3.40825 | -2.91749 | 2.67420 | 0.42633 |
| 2012-2013 | -8.91359 | 1.33536 | 0.65117 | -1.60121 | -1.70574 |
| 2013-2014 | 9.88713 | 0.57776 | -0.83562 | -0.63355 | 0.54320 |
| 2014-2015 | -9.62607 | 7.15842 | -0.63706 | -1.22840 | -0.54008 |
| 2015-2016 | -5.38153 | 11.95988 | -3.55285 | -0.69589 | 0.04232 |
| 2016-2017 | -2.60380 | 13.35481 | -2.59877 | -0.43236 | 1.97229 |
| 2017-2018 | 0.20754 | 12.15004 | -0.51900 | -1.13725 | 4.64763 |
| 2018-2019 | -7.29918 | 11.57011 | -2.69461 | 1.10532 | 5.31803 |
| 1990-2019 | -119.77421 | 176.30903 | 228.18391 | 46.85682 | 88.21193 |

**Appendix C**

Transport decomposition and factor variations.

| Time | $\Delta SEC_{ES_{\text{l }effect}}$ | $\Delta SEC_{EI_{\text{l }effect}}$ | $\Delta SEC_{TI_{\text{l }effect}}$ | $\Delta SEC_{TP_{\text{l }effect}}$ | CV |
| --- | --- | --- | --- | --- | --- |
| 1990-1991 | 1.37809 | 0.92885 | -2.26927 | 3.33395 | 0.84291 |
| 1991-1992 | 9.62310 | 4.66007 | -7.33991 | 9.63807 | 4.98824 |
| 1992-1993 | 0.85537 | 4.40632 | 1.35625 | 3.64499 | 7.55397 |
| 1993-1994 | 0.32569 | 6.22567 | 11.19522 | -11.19522 | 9.19181 |
| 1994-1995 | 1.58811 | 4.65157 | -4.80821 | 3.43690 | 10.40890 |
| 1995-1996 | -2.79480 | 20.62527 | -10.98820 | 3.53665 | 13.00363 |
| 1996-1997 | 4.43029 | -3.57171 | -27.33934 | 27.33934 | 13.21828 |
| 1997-1998 | -0.67330 | 0.04067 | 3.99860 | 0.76889 | 14.25199 |
| 1998-1999 | 6.11836 | 0.39907 | 0.87849 | 3.97643 | 17.09508 |
| 1999-2000 | -0.82294 | -39.99310 | 67.27357 | -16.67749 | 19.54009 |
| 2000-2001 | -1.81809 | -3.03334 | -0.89926 | 3.72957 | 19.03481 |
| 2001-2002 | -3.89093 | 4.18193 | -36.47567 | 34.69758 | 18.66304 |
| 2002-2003 | -1.59193 | 6.87862 | -5.79107 | 3.72653 | 19.46857 |
| 2003-2004 | -7.35787 | 26.10625 | -10.69298 | 2.28271 | 22.05310 |
| 2004-2005 | -4.05825 | 26.22759 | -10.94662 | 4.80309 | 26.05955 |
| 2005-2006 | -22.77944 | 13.12481 | -20.38144 | 18.31969 | 23.13046 |
| 2006-2007 | -6.86168 | 8.98573 | 9.04504 | -6.55530 | 24.28391 |
| 2007-2008 | 17.96797 | 18.44844 | -8.58377 | 9.60664 | 33.64373 |
| 2008-2009 | 8.57387 | -23.13328 | 11.44167 | -0.84731 | 32.65246 |
| 2009-2010 | -2.99541 | 7.85430 | -5.83959 | 6.71894 | 34.08702 |
| 2010-2011 | 6.93361 | 3.17879 | 0.70655 | -3.42897 | 35.93452 |
| 2011-2012 | 3.53025 | 5.78547 | -4.49748 | 6.19725 | 38.68839 |
| 2012-2013 | 2.04506 | 0.04806 | 3.67799 | -2.71198 | 39.45317 |
| 2013-2014 | 6.85281 | -1.98327 | -22.48005 | 22.08842 | 40.57265 |
| 2014-2015 | -1.03797 | 11.60975 | -0.85927 | 3.48009 | 43.87080 |
| 2015-2016 | 11.75248 | 21.90420 | -12.00381 | 12.87537 | 52.50286 |
| 2016-2017 | 2.41224 | 35.41457 | -18.65591 | 15.72183 | 61.22604 |
| 2017-2018 | -0.45457 | 46.18769 | -22.10216 | 9.40878 | 69.48598 |
| 2018-2019 | 7.87325 | 22.74291 | -52.59975 | 62.98807 | 79.73709 |
| 1990-2019 | 34.23315 | 211.48351 | -150.63720 | 223.86891 | 159.47419 |
